# Function of miR-212 as a tumor suppressor in thyroid cancer by targeting SIRT1

**DOI:** 10.3892/or.2021.8173

**Published:** 2021-08-24

**Authors:** Dandan Li, Lin Bai, Tongtong Wang, Qian Xie, Minglong Chen, Yantao Fu, Qiang Wen

Oncol Rep 39: 695-702, 2018; DOI: 10.3892/or.2017.6119

Following the publication of this paper, it was drawn to the Editors' attention by a concerned reader that the tumor images shown in Fig. 6B bore unexpected similarities to data appearing in different form in other articles by different authors. In addition, there were potential anomalies associated with the cell migration assay data shown in Fig. 2E. Owing to the fact that some of the contentious data in the above article had already been published elsewhere, or were already under consideration for publication, prior to its submission to *Oncology Reports*, the Editor has decided that this paper should be retracted from the Journal. The authors agree with the decision to retract the paper. The Editor apologizes to the readership for any inconvenience caused.

